# Retarded Growth

**Published:** 1883-08

**Authors:** 


### EDITORIAL.

### Retarded Growth.

A young practitioner presents a case about which he is quite anxious. It consists in the tardy growth of a second permanent superior right bicuspid. It is in the mouth of a young lady seventeen years of age. The temporary molar remained until she was thirteen. Soon after its displacement the bicuspid made its appearance and slowly came with its crown surface, just through the gum. Within a short time its growth seemed to be arrested, and it has remained to the present time. The question now is, can the tooth be made to assume its normal size and position ? That, of course, cannot be decided without an effort. It is probably kept down by the first bicuspid and the first molar.

If this is the case, and either one is decayed next to the tardy tooth, relief from pressure may be obtained by cutting. If there is no decay, these teeth should be pressed apart by some elastic substance sufficiently to accommodate the tardy tooth. In some instances the relief from such pressure is followed by prompt growth of the tooth. In some cases, however, such growth does not occur. It is practicable in such cases to apply traction with a rubber band and the properly adjusted spring. This will only be successful in some cases. Care should be exercised by this application of force, lest the tooth be drawn from its socket. The force should be regulated, as the tooth will bear it. This is one of the anomalies that require much judgment and attention for the attainment of the best results. If anything has occurred by which the remaining portion of the germ of the tooth has been destroyed, the case is hopeless through any treatment that might be employed.

